# Genetic regulation of *MUC1* alternative splicing in human tissues

**DOI:** 10.1038/sj.bjc.6604617

**Published:** 2008-08-26

**Authors:** W Ng, A X W Loh, A S Teixeira, S P Pereira, D M Swallow

**Affiliations:** 1 Research Department of Genetics, Evolution and Environment, University College London, Wolfson House, 4 Stephenson Way, London NW1 2HE, UK; 2 Division of Medicine, Institute of Hepatology, Royal Free & University College London Medical School, London, UK; 3 University Department of Paediatrics, John Radcliffe Hospital, Oxford, UK

**Keywords:** MUC1, splicing, polymorphism, transcript, gene regulation

## Abstract

The membrane mucin MUC1 is aberrantly expressed in a variety of cancers, and in stomach, it is a ligand for *Helicobacter pylori* where it plays a role in gastric carcinogenesis. Splicing variation, leading to a 9-amino acid insertion in the signal peptide region, was proposed to be because of a single-nucleotide polymorphism (rs4072037) at the 5′ end of exon 2, but is also reported to be cancer-associated. However, the effect of rs4072037 on this splicing event in healthy non-cancer tissues and on the additional spliceoforms of *MUC1*, including those lacking the polymorphic tandem repeat (TR) domain, has never been investigated. Here we show that in both foetal and adult tissues of known genotype, there is clear evidence for the role of rs4072037 in controlling alternative splicing of the 5′ exon 2 region of both full-length transcripts and those lacking the TR domain. Although there is some evidence for additional genetic and epigenetic influences, there is no indication of an effect of the TR domain on the proportions of the spliceoforms. In conclusion, over-representation of certain transcripts in tumour material cannot be evaluated without information on the SNP genotype as well.

MUC1 is a highly polymorphic membrane-associated mucin that is often aberrantly expressed in cancer (Taylor-Papadimitriou *et al*, 2002). It has a centrally located tandem repeat (TR) domain (Lan *et al*, 1985; Gendler *et al*, 1987, 1988, 1990; Swallow *et al*, 1987; Hareuveni *et al*, 1990) comprised of 20–120 or more repeat units of 60 nucleotides, which encode 20 amino acids. The repeating units include several serine and threonine residues, which carry most of the glycosylation, and this glycosylation, as well as the general pattern of expression, is altered in cancerous cells (Taylor-Papadimitriou *et al*, 2002). Susceptibility to *Helicobacter pylori* gastritis and to gastric cancer appears to be associated with *MUC1* allele length (Carvalho *et al*, 1997; Silva *et al*, 2001, 2003; Vinall *et al*, 2002). MUC1 binds to *H. pylori* (Linden *et al*, 2004; McGuckin *et al*, 2007), *Muc1* knockout mice are susceptible to *H. pylori* gastritis (McGuckin *et al*, 2007) and there is an altered pattern of expression of MUC1 in *H. pylori* gastritis (Vinall *et al*, 2002). The observations that MUC1 plays a role in the progression to gastric cancer highlight the importance of understanding all the aspects of the normal variation of this gene.

During the course of various cancer-related studies, several variant *MUC1* transcripts were reported. Early cloning experiments revealed the presence of transcripts (**a** and **b**) with an alternative 27 bp intron retention event at the start of exon 2 (Ligtenberg *et al*, 1990). Although a genetic basis for this variable splicing event was first suggested by Ligtenberg *et al* (1991), who used cancer cell lines, the longer **a** transcript was also reported to be more abundant in cancer tissues (Weiss *et al*, 1996; Obermair *et al*, 2001, 2002; Schmid *et al*, 2002, 2003) and to have potential prognostic value. Therefore, the function of the genetic polymorphism implicated (rs4072037 G/A, located at nucleotide position 8 of exon 2 of the **b** variant) was left in some doubt. An additional alternative splicing event is known to occur at the 5′ end of exon 2, which generates two minor transcripts denoted as **c** and **d**, and was first reported by Obermair *et al* (2001). Variants **c** and **d,** respectively, have 9 and 27 fewer nucleotides, at the start of exon 2, than the **b** transcript. The splice variants observed in breast (Schmid *et al*, 2002, 2003), cervical (Obermair *et al*, 2001) and ovarian tumours (Obermair *et al*, 2002) were suggested to be associated with transcripts **b** and **a**, respectively, and thus are possibly also under the influence of rs4072037. These alternative splicing events lie within the signal peptide domain and are very close to two signal peptide cleavage sites that have been observed experimentally in **b** variant (Parry *et al*, 2001).

*MUC1* is also known to have alternative spliceoforms that lack the TR domain (Zrihan-Licht *et al*, 1994a, 1994b; Baruch *et al*, 1997, 1999; Obermair *et al*, 2001, 2002). Each of these transcripts is generated through the use of one additional 5′ splice site and one of three different 3′ splice sites in the sequence, which flank the TR domain in exon 2. *MUC1*/Z is the longest transcript, followed by *MUC1*/Y, which is 54 bases shorter, followed by *MUC1*/X, which is a further 19 bases shorter, using the nomenclature adopted by Obermair *et al* (2002). The three transcripts and an additional form called *MUC1*/Y alt, some of which are likely to be functional (Levitin *et al*, 2005), have been detected in breast tumours (Baruch *et al*, 1997, 1999; Hartman *et al*, 1999; Obermair *et al*, 2001), cervical tumours (Obermair *et al*, 2001) and ovarian tumours (Obermair *et al*, 2002).

The aim of this study was to investigate the role of rs4072037 in relation to the alternative splicing events of *MUC1*, in both TR-containing and TR-negative transcripts, for a range of normal non-cancer tissues, to help evaluate published results and as a platform for future cancer studies. Foetal samples were used to obtain several tissues from a single individual as well as the same tissue from many individuals. In addition, a number of adult gastric and duodenal samples and cancer cell lines were examined. As the TR domain makes up a significant proportion of the transcript for *MUC1* and is known to show considerable length variation in different individuals, this might affect the rate of transcript synthesis or transcript stability. The TR lengths of *MUC1* were therefore examined in the same set of foetal samples.

## Materials and methods

### Samples

Foetal tissues were obtained from the MRC Tissue Bank and from the adult tissues from patients undergoing clinically indicated gastroscopy at University College London Hospitals NHS Foundation Trust (Joint UCL/UCLH Committees on the Ethics of Human Research approvals 04/0011 and 01/0237). Genomic DNA and RNA from nine cancer cell lines were also tested: lung cancer cell lines A427, A549, NCI-H522, NCI-H358, NCI-H441, NCI-H23, NCI-H460, laryngeal carcinoma and HEP-2, and were kindly supplied by Dr Jeremy Hull, University Department of Paediatrics, John Radcliffe Hospital, Oxford.

### RNA preparation

Tissues were stored at −70°C. Frozen tissue (25–50 mg) was crushed into a fine powder with a pestle and mortar, followed by RNA extraction using RNA-Bee™ Biogenesis Ltd, Poole, UK, according to the manufacturer's protocol. The total RNA was re-suspended in RNase-free water, quantified using a spectrophotometer and the quality checked on 1% agarose gels.

Southern blot analyses were performed as previously described (Fowler *et al*, 2003).

### DNA preparation

For PCR-based genotyping, genomic DNA was retrieved from the residue and organic phase of the RNA extraction mixture, after removal of the aqueous phase, by adding a back extraction buffer (4 M guanidine thiocyanate, 50 mM Na citrate, 1 M Tris, pH 10.5), followed by DNA precipitation using isopropanol, as described in the manufacturer's instructions. Higher quality tissue DNA, used for Southern blotting, was prepared using the Puregene® DNA Purification Kit (Gentra Systems, Minneapolis, MN, USA): 10–20 mg tissue was crushed in liquid nitrogen with a pestle and mortar and the DNA extracted according to the manufacturer's instructions.

### cDNA synthesis

Complementary DNA was synthesised with M-MLV Reverse Transcriptase (Invitrogen, Paisley, Scotland) using 200 or 400 ng of total RNA, 0.2 mM of each dNTP, 0.25 *μ*g random hexamers and 20 U of RNAase inhibitor (RNaseOUT™, Invitrogen) in 20 *μ*l. For PCRs, a 1/10 dilution of the cDNA was used. In each case, control transcriptions were run with no reverse transcriptase; no-template blanks were also included in the PCRs as negative controls. Primers from a non-polymorphic region of *MUC1* and from the ribosomal S14 gene (*RPS14*) were used as controls for template quality.

Oligonucleotide primers were purchased from Sigma-Genosys, Haverhill, UK. Thermocycling was conducted on an MJ Research PTC-200 Peltier Thermal Cycler (Genetic Research Instrumentation, Braintree, UK). Primer sequences and cycling conditions are shown in Supplementary Table 1.

For rs4072037 typing, the primers 1946 AGS and Cy5-labelled Exon2AS (see Supplementary Table 1) were used to generate a 260 bp product, followed by restriction enzyme digestion using *AlwN1*, for which the sequence cuts when there is an A allele and fails to cut if there is a G allele, as was done earlier (Fowler *et al*, 2003). Samples with no uncut product were interpreted as AA and no cut product, as GG, assuming no silent alleles.

Electrophoresis was conducted on 4.8% acrylamide (19 : 1) UltraPure™ Sequagel gels using an ALF express™ sequencer (Amersham Pharmacia, Little Chalfont, UK). PCR products were run with molecular size markers mixed with the PCR product and loading buffer. The same technique was used for transcript analyses. The relative peak heights were also recorded to semi-quantify the relative amount of **a** and **b** transcripts.

PHASE (version 2.1), which uses a Bayesian statistical method for haplotype construction (Stephens *et al*, 2001), was downloaded from http://stephenslab.uchicago.edu/software.html.

## Results

### Relationship of splicing with genotype: variable exon 2 spliceoforms

**Figure 1:**
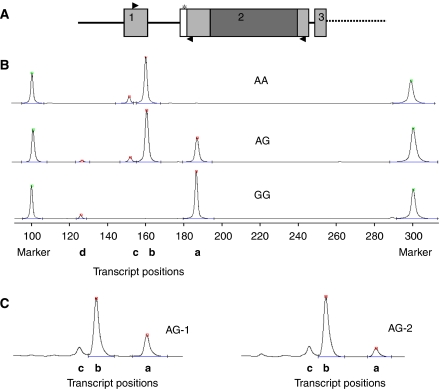
(**A**) Diagram of the start of *MUC1* showing the positions of the PCR primers in relation to rs4072037 (asterisked). The variable extension of exon 2 is shown in white. Tandem repeat domain is shown in dark grey. The positions of the single-sense primer and two antisense primers are shown with arrows. The antisense primer downstream of the TR domain is used for amplifying the components lacking the TR domain. (**B**) ALF Express trace of the RT–PCR from three individuals with different rs4072037 genotypes, using the primer in exon 1 in combination with the primer upstream of the TR domain. The positions of the four transcripts, **a**, **b**, **c** and **d**, as well as the 100 and 300 bp markers are indicated. (**C**) ALF Express traces from two AG heterozygous individuals, one of whom shows very little **a** transcript. Samples from foetal lung; complete traces are shown in Supplementary Figure 1.

All the samples were first typed for rs4072037. Corresponding cDNA from 68 foetuses (total of 120 tissue samples), 36 adults (21 gastric and 15 duodenal samples of which 13 and 9, respectively, had normal histology and the rest had various degrees of inflammation) as well as the nine cancer cell lines were amplified using primers to detect the variable splicing at the 5′ end of exon 2 (Figure 1A).

Up to four possible transcripts, **a**, **b**, **c** and **d**, could be detected in a single sample. Figure 1B shows PCR products from three representative individuals of AA, AG and GG genotypes. The results are summarised in Table 1.

There was a clear correlation between the transcripts observed and the rs4072037 genotype. In all cases where the rs4072037 G allele was present, **a** transcripts were present in the RNA, and when the rs4072037 A allele was present, there were **b** transcripts. In the AA homozygotes, there was no **a** transcript, and in most GG homozygotes, there was no **b** transcript, although in two GG foetal lungs and one GG foetal stomach, one GG adult stomach and one GG adult duodenum, a trace amount of the **b** variant was detected. In the AG heterozygotes, both **b** and **a** transcripts were observed, though **b** was usually more prominent and the proportions of **a** and **b** were variable (Figure 1C).

Expression of the two minor transcripts, **c** and **d**, was also associated with the rs4072037 allele. When detected, the smallest transcript **d** was always associated with G allele, which also produces the **a** transcript. The **c** transcript on the other hand was found with the A allele, which also generates the **b** transcript.

### Spliceoforms lacking the TR domain

**Figure 2:**
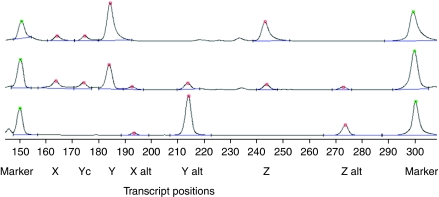
Variant transcripts lacking the TR domain, detected using primers Cy5 M1ProF4 and MUC1X2R. The positions of the transcripts X, Yc, X alt, Y alt, Z, Z alt, and the 150 and 300 bp markers are indicated. Samples from AA, AG and GG individuals.

The expression of the ‘non-TR’ transcripts was examined using the foetal tissues and with the same protocol with the same exon 1 primer and a reverse primer located at the 3′ end of exon 2 (Figure 1), except that two extra rounds of PCR amplification and double the cDNA concentration were usually required to detect these components. Up to seven different transcripts were detected (Figure 2). A summary of the results is shown in Table 2. On the basis of their sizes, the non-TR transcripts were identified as the previously reported *MUC1*/X, *MUC1*/Y, *MUC1*/Z, as well as *MUC1*/Y alt (Obermair *et al*, 2001), the non-TR transcript analogous to **a**. Two additional components were 27 bases larger than *MUC1*/X and *MUC1*/Z, respectively, and thus are expected to be alt or **a**-like variants of transcripts X and Z, respectively, and were thus named *MUC1*/X alt and *MUC1*/Z alt. *MUC1Yc* was so named because it is 9 bases shorter than the *MUC1*/Y variant and thus likely to correspond to a non-TR version of **c**.

A clear genotype-associated pattern of expression for these variants was observed (Table 2). In rs40723037 AA homozygotes, only the non-alt variants were present, whereas in rs40723037 GG homozygotes, only the alt variants were observed. In the AG heterozygotes, both the alt and non-alt forms were detected.

### Variability of proportions of the major transcripts **a** and **b**

The fact that in the rs4072037 heterozygotes, the peak heights of the **a** transcript were almost always lower than the **b** transcript (Figure 1B and C) is probably partly a reflection of the relative signal detection by the ALF Express™ machine and may also reflect the PCR of the different sized fragments (**a** being 27 nucleotides longer than **b**). However, the relative levels of the two transcripts (Figure 1C), though variable, were reproducible for a particular sample. Although measurements using the ALF Express could at best be considered semi-quantitative, peak heights were recorded to evaluate this variability in relation to tissue source and individual.

### Tissue differences

Consideration of all heterozygous samples of a particular tissue showed that the **a**/**b** ratios were not normally distributed, particularly in the case of the liver and trachea. The median values obtained for each of the tissues were 0.47 (range 0.06–0.82) for lung, 0.49 (range 0.12–0.64) for stomach, 0.7 (range 0.08–1.33) for liver and 0.26 (range 0.04–0.35) for trachea, and the data sets were compared using the non-parametric Mann–Whitney test. There was a significant difference in transcript ratio between the liver and trachea (*P*=0.0128) and between each of these and the lung (*P*=0.011) (*P*=0.0047) or stomach (*P*=0.0053) (*P*=0.0016). There was no significant correlation of transcript proportions with gestational age, although only a relatively small range of gestational ages was represented within the sample set (11–20.4 weeks).

### Inter-individual differences: allelic origin of the transcripts

The **a**/**b** transcript ratio in three of the heterozygotes was particularly imbalanced (Figure 1C, individual 2). In one of these three cases, two tissues were available from the same individual, and both had a very low expression of **a**. To interpret the source of this asymmetry, we determined the allelic origin of the **a** and **b** transcripts for this individual, by digesting the cDNA with *AlwNI,* the enzyme used for rs4072037 genotyping in genomic DNA. The enzyme cuts the rs4072037 A allele, which is expected to be present in the **b** transcript, whereas the **a** transcript is expected to have the rs4072037 G allele and would not be cut. Samples from both the trachea and lung were tested. A ‘typical’ rs4072037 AG heterozygote and an rs4072037 AA and rs4072037 GG homozygote were used as controls. The results (Supplementary Figure 1) show that the **G** allele is completely cut by the enzyme and thus does not contribute to the **b** transcript in either heterozygote, and implies that the G allele is less stable or poorly transcribed in the individual with very low amounts of **a** transcript.

### Possible genetic origin of inter-individual differences

The detection of dramatically lower amounts of **a** transcript in two tissues from the same individual and the non-normal distributions of the **a**/**b** transcript ratios suggested the possibility of a genetic origin for some of the differences in transcript ratio. In support of this, ratios obtained from the lung and stomach from the same individuals showed a significant correlation (*P*=0.05, data not shown).

**Figure 3:**
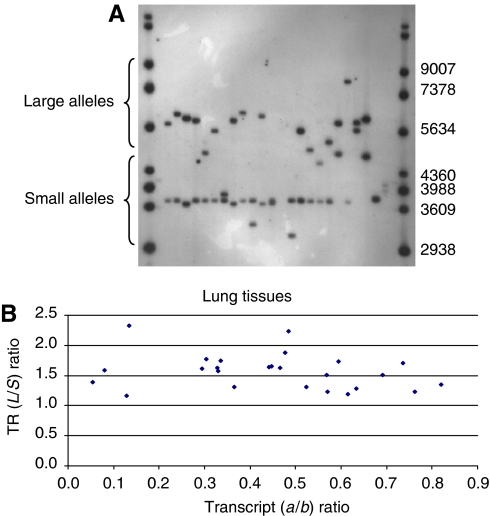
(**A**) *MUC1* VNTR polymorphism detected on a Southern blot. The band sizes for the Raoul ladder size markers in the first and last tracks are shown on the right. Grouping of long (L) and short alleles (S) used for haplotype analysis is shown. (**B**) *MUC1*a/b transcript ratio (lung samples) plotted against *MUC1TR* length ratio. Similar results were obtained for stomach (data not shown).

The possibility that the rate of transcript synthesis or transcript stability is affected by the variable lengths of the TR domains of *MUC1* was therefore examined by testing the TR polymorphism in the same set of foetal samples and comparing allele length ratios with transcript ratios for the lung and stomach, the two tissues for which the most samples were tested. Figure 3A shows an autoradiograph of a typical Southern blot.

The haplotypes comprised of rs4072037 (G or A) and the TR length, binned as long (L) and short (S) intronic, were inferred using PHASE. Approximately 90% of the chromosomes have non-recombinant haplotypes, namely, A-S (58%) or G-L (33%) with only 5% AL and 4% GS, consistent with what was found previously in Europeans (Fowler *et al*, 2003; Teixeira, 2005). Thus, one can infer that in the majority of rs4072037 AG heterozygotes, the **b** transcript encoded by the A allele carries a short TR array, whilereas the **a** transcript encoded by the G allele carries a long array. Less than 0.2% double heterozygotes (5% × 4%) are expected to have the converse arrangement.

Figure 3B shows the TR length ratios (length of the long TR domain, typically rs4072037 G allele carrying haplotype over the short TR domain, typically the rs4072037 A allele carrying haplotype, see above) plotted against the **a**/**b** transcript ratios present in the lung. There was no statistically significant correlation for either the lung or the stomach. There was also no significant correlation when differences in absolute length were plotted against the transcript ratio (data not shown).

### Data mining

All human exon 1 and exon 2 boundary containing *MUC1* transcripts, annotated on the UCSC Browser as of June 2007, were retrieved from the GenEMBL database. Where available, all original literature references were examined to confirm that the full sequence was taken from the clones reported and checked to determine whether any composite sequences were included in the list. For 15 of 37 annotated *MUC1* sequences, literature references were available. In 14 of the 15 referenced sequences, appropriate sequencing appeared to have been done. In one case, the transcript submitted was declared as a composite sequence (Ligtenberg *et al*, 1990).

Sequences upstream of the TR domain in exon 2 were aligned with ClustalW. Four classes of transcripts were observed, which were named for convenience as **a**, **b**, **c** and **d**. As most were either short transcripts or incomplete sequences, it was not formally possible to determine how many came from transcripts that contain a full TR domain. The **a** variant is represented by eight transcripts and the **b** transcripts by 26 sequences (see Supplementary information for list), 13 of which were from HeLa cells submitted from one laboratory (Zhang and Lu, 2003, database submission only).

All 26 **b** transcripts have the rs4072037 A allele. Although five of eight annotated **a** transcripts have the rs4072037 G allele, the other three transcripts carried the A allele. All three (M32738, AY327587 and S81781) were from cancer-derived material, but it is noteworthy that one was from the composite sequence (Ligtenberg *et al*, 1990), and one has no associated literature reference. Two **c** transcripts (AF125525 and Z17325) and one **d** transcript (Z17325) were also identified but the rs4072037 A/G SNP is not included within the shorter exonic sequence of **c** and **d** so that genotype information cannot be obtained.

### Splice-prediction programs

A number of bioinformatic prediction tools (SplicePredictor: http://deepc2.psi.iastate.edu/cgi-bin/sp.cgi, GeneSplicer: http://www.tigr.org/tdb/GeneSplicer/gene_spl.html, Berkeley Drosophila Genome Project: http://www.fruitfly.org/ and NetGene2: http://www.cbs.dtu.dk/services/NetGene2/) were also used to examine which if any of the alternative 3′ splice sites was predicted by the programs. The 3′ splice site for the **b** transcript was not predicted by any of these four programs. SplicePredictor, however, predicted the 3′ splice site that is used for the **a** transcript (regardless of nucleotide at rs4072037). This is consistent with the calculated strength of the splice sites based on the algorithm of Shapiro and Senapathy (1987), which suggested that the strength of the 3′ splice site used by the **a** transcript is significantly stronger than the site used for the **b** transcript.

The results of the analysis using the program Splice Sequences Finder version 2.2 (http://www.umd.be/SSF), which predicts exonic motifs, showed that three exonic splicing enhancer motifs and one exonic splicing silencer motif overlap the rs4072037 SNP in the presence of either the A or the G alleles. However, the two allelic variants were predicted to bind different proteins.

## Discussion

This is the first large scale study that shows a clear association of alternative splicing of the 5′ exon 2 region of *MUC1* with the rs4072037 A/G SNP. Both experimental and database trawling show that there is a strong association between transcript type and exon 2 allele status, implying that SNP rs4072037 controls splice acceptor usage as originally predicted from data obtained using cancer cell lines (Ligtenberg *et al*, 1991). In addition to the major spliceoforms, the polymorphism affects all the minor ones, including ones with additional deletions in exon 2 (**c** and **d**) and those without a TR domain, the latter association being described here for the first time. Our study confirms that these components are present as a minor fraction of the *MUC1* transcripts in ‘normal’ as well as cancer tissues. The alternative splicing at the start of exon 2 is independent of alternative splicing of the TR region, which is not controlled by rs4072037. While this work was in progress, an association of rs4072037 with the **a**, **b**, **c** and **d** transcripts was also reported in adult corneal tissue (Imbert *et al*, 2006), confirming the effect in another tissue. These authors did not, however, test genomic DNA but rather deduced genotype from cDNA.

Analysis of haplotypes 310 000 bp downstream and 330 000 bp upstream of *MUC1TR* using CEPH trio data shows a number of SNPs in very strong linkage disequilibrium with rs4072037 (data not shown). However, none were completely associated with rs4072037. In addition, extensive re-sequencing (NIEHS (http://egp.gs.washington.edu/) and A Teixeira and DM Swallow, unpublished) failed to identify any other intragenic SNPs, notably there being none in intron 1 that might have been responsible for this splicing event. Indeed no other common SNPs have been identified within the gene, showing that rs4072037 must be directly responsible for the splicing polymorphism.

However, this splicing polymorphism was not predicted by any of the exon prediction programs currently available. Indeed for both alleles, the longer transcript was predicted. On the other hand, the A-to-G substitution is predicted to alter protein binding in Splice Sequences Finder version 2.2. The secondary structure of the pre-mRNA is also predicted to be different, where only the G allele forms a physiologically stable stem loop structure (as predicted by the mfold program; http://frontend.bioinfo.rpi.edu/applications/mfold/cgi-bin/rna-form1.cgi) (Mathews *et al*, 1999; Zuker, 2003). Indeed the possible importance of this difference was noted previously (Ligtenberg *et al*, 1991). Whatever the mechanism, there appears to be some leakiness in the control: small amounts of the **b** transcript can be found in a few of the normal tissues from GG homozygotes, and in the sequences submitted to databases, a few **a** transcripts from cancer tissues were found that carry the A allele. The proportions of the transcripts are variable in heterozygotes, and this is not attributable to this leakiness as shown by the restriction enzyme digestion experiments. It seems to reflect cell-to-cell (and thus tissue-to-tissue) as well as person-to-person differences in allelic expression (as opposed to splicing), which may be epigenetic or genetic in origin. Comparison of the relative differences in allelic expression with relative length of the TR domain suggests that the genetically determined differences in transcript length do not significantly affect transcript quantity.

Putting together all these observations, it can be concluded that none of these transcripts are tumour-specific. Their over-representation in tumour material cannot really be evaluated without information on the SNP genotype as well. It is possible that inflammation and cancer affect the relative expression of the alleles and/or splicing. However, it is also possible that the genotypes were unevenly represented in the cohorts under study. In most studies, the **a** transcript (normally encoded by the G allele) was more abundant. Studies on gastric cancer and gastritis (Carvalho *et al*, 1997; Vinall *et al*, 2002) have shown that *MUC1TR S* alleles are over-represented, and the A allele (not the G allele) is normally associated with short TR alleles (Pratt *et al*, 1996; Vinall *et al*, 2002). However, in our own work, we have shown that it is in fact the recombinant GS alleles that are over-represented in gastric cancer and gastritis (A Teixeira *et al*, unpublished). Unfortunately, in most of the studies that suggest an association of tumorigenesis with the **a** transcript, data for neither the SNP nor the TR length were collected. Thus, it is possible that the associated rs4072037 G allele is often present as a recombinant haplotype with the short TR domain. These recombinant haplotypes are present at a reasonable frequency in the population (7–10%).

Interestingly, in one paper (Schmid *et al*, 2002) where genotyping was done, substantial quantities of **a** transcript were detected in three cell lines, which were homozygous AA for rs4072037. This is suggestive of secondary ‘leakiness’ rather than allelic differences in expression and could be a genuine cancer-related change in splicing, which is consistent with the finding of some tumour-derived A allele **a** transcripts on the databases. These observations, together with the study described here, also indicate that transcript phenotype cannot be used as a reliable surrogate for genotype. It had been our intention to examine these splicing events in relation to *H. pylori* gastritis. It is clear, however, from the work reported here that there are too many variables to address this question without having a very large cohort of samples and better quantitative methods.

**Figure 4:**
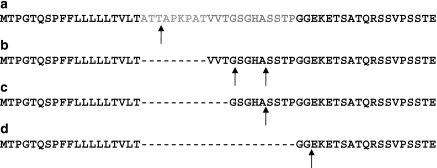
Amino-acid sequences of the N-terminal ends of the **a**, **b**, **c**, and **d** encoded peptides and positions of the observed and/or predicted peptide cleavage. The sequence that undergoes alternative splicing is shown in grey on the **a** transcript. The upward arrows show the positions of predicted, or in the case of the **b** transcript the experimentally observed, peptide cleavage sites.

It should be emphasised that the allelic variation of rs4072037 polymorphism may affect the function of MUC1 protein. The alternative splicing event occurs within the signal peptide of MUC1 in the vicinity of the known proteolytic cleavage sites found for the b variant (Parry *et al*, 2001). Although there is no experimental evidence for the **a** variant, SignalP 3.0 site (http://www.cbs.dtu.dk/services/SignalP/), which accurately predicts cleavage of the **b** variant, suggests that the signal peptide for the **a** variant will end at residue 23 from the start of translation, and an above threshold signal peptide cleavage site was predicted to lie between the T and A residues at positions 22 and 23, respectively. This is within the region that contains the inserted amino-acid sequence. The predictions for all the 5′ transcript variants are shown in Figure 4.

The normal MUC1 biosynthesis pathway and glycosylation include targeting of the mature protein to the apical surfaces of epithelial cells, and we have previously shown that this is altered in *H. pylori* gastritis (Vinall *et al*, 2002). The alternative splicing events within the signal peptide sequence domain could possibly lead to differences in cellular trafficking, as observed in interleukin-15 (Kurys *et al*, 2000), altering the localisation of the mature protein. If, however, variation in the signal peptide does affect apical targeting, in either the normal or diseased mucosa, the effect is not ‘all or nothing’ because apical staining is seen in the normal mucosa of individuals of all three genotypes and no staining was seen in any of the gastritis specimens regardless of genotype (LE Vinall *et al*, unpublished).

## Figures and Tables

**Table 1:** Summary of splice variants **a**, **b**, **c** and **d** observed in foetal and adult tissues and cell line samples from individuals of different genotypes for rs4072037

**Genotypes**	**AA**	**AG**	**GG**
*Splice variants present*			
*Foetal lung*			
Total no.	22	23	5
**a**/**d**			3
**b**/**c**	21		
**a**/**b**/**c**/**d**		23	
**a**/trace-**b**			2^a^
			
*Foetal stomach*			
Total no.	19	18	5
**a**/**d**			4
**b**/**c**	19		
**a**/**b**/**c**/**d**		18	
**a**/trace-**b**			1^a^
			
*Foetal liver*			
Total no.	2	6	
**b**/**c**	2		
**a**/**b**/**c**/**d**		5	
**a**/**b**/**c**			
**a**/**b**/**d**		1	
			
*Foetal trachea*			
Total no.	3	7	1
**a**/**d**			1
**b**/**c**	3		
**a**/**b**/**c**/**d**		4	
**a**/**b**/**c**		3	
			
*Adult stomach*			
Total no.	6	11	4
**a**/**d**			3
**b**/**c**	3		
**b**	2		
**a**/**b**		1	
**a**/trace-**b**			1^a^
**a**/**b**/**c**/**d**		10	
			
*Adult duodenum*			
Total no.	1	8	6
**a**/**d**			3
**a**			2
**b**	1		
**a**/**b**/**c**/**d**		6	
**a**/**b**/**c**		2	
**a**/trace-**b**			1^a^
			
*Cell lines*			
Total no.	3	4	2
**a**/**d**			2
**b**/**c**	3		
**a**/**b**/**c**/**d**		4	

a Samples that contain low amounts of transcripts not usually associated with their genotype.

**Table 2:** *MUC1* ‘non-TR’ transcripts observed in the foetal sample series, subdivided according to genotype, and tissue

**Genotype rs4072037**							**AA**			**AG**			**GG**	
**Total individuals**							25			31			7	
**Total tissues**							42			48			10	
**Spliceoforms**														

**X**	**Yc**	**Y**	**Z**	**X alt**	**Y alt**	**Z alt**	**Lung**	**Stomach**	**Liver**	**Lung**	**Stomach**	**Liver**	**Lung**	**Stomach**
√	√	√	√				10	8		1				
√	√	√								1				
	√	√	√				3							
√		√	√				3	4						
√		√					2			1				
	√	√					2				1			
		√	√				3	1				1		
		√					3	2	1		1	1		
√	√	√	√	√	√	√				2	2			
√	√	√	√		√	√				1	2			
√	√	√	√		√					1	2			
√	√	√	√		√						3	1		
√	√	√	√	√	√					1				
√		√		√	√	√				3				
	√	√	√		√	√				1	1			
√	√	√			√							1		
√		√			√	√				1				
√		√	√		√	√				3	2			
√		√	√		√					1				
		√	√		√	√				1				
	√	√			√	√				3				
		√		√	√	√				1				
		√			√	√				1	1	1		
	√					√						1		
		√			√					3	1			
				√	√	√							3	1
				√	√								1	
					√	√							1	3
					√								1	
Total							26	15	1	26	16	6	6	4

Presence of the ‘alt’ splice variants is correlated with the presence of the rs4072037 G, and that of X, Y and Z transcripts is correlated with the A allele.
