# Supplementary material for: Genetic regulation of MUC1 alternative splicing in human tissues
Source: Br J Cancer. 2008 Aug 26;99(6):978–85. doi: 10.1038/sj.bjc.6604617 (PMC2538764; doi:10.1038/sj.bjc.6604617)
Supplement: Supplementary Figure 1 [file 6604617x1.doc]

Supplementary Figure 1


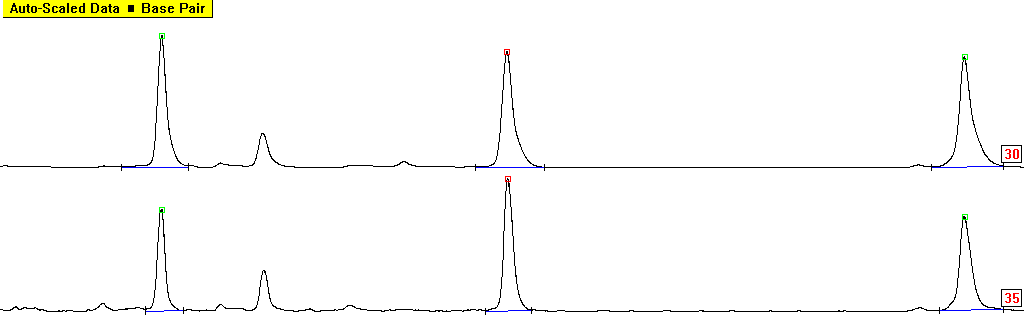


**GG**


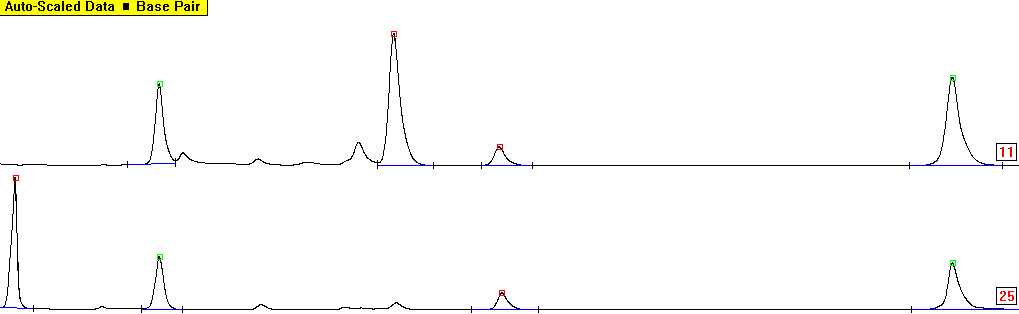

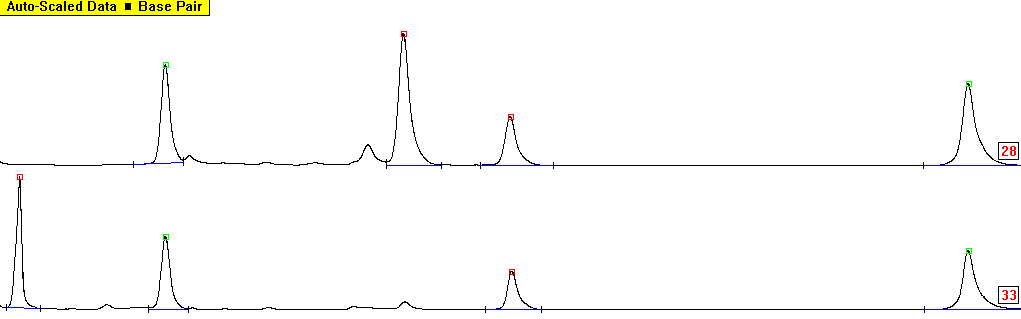

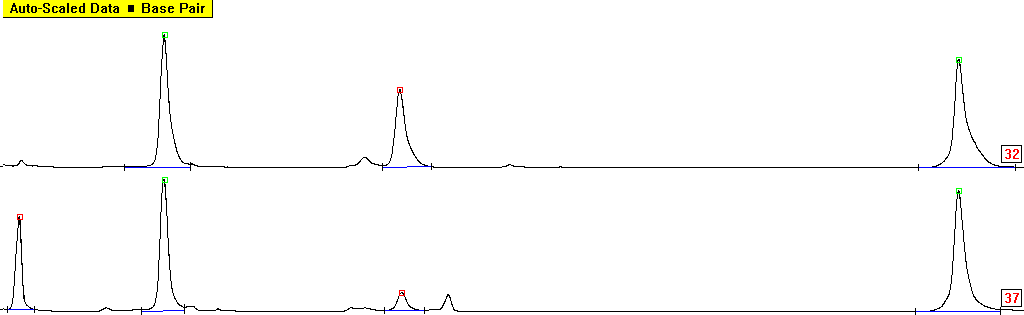


**AA**

**AG**

**AG**

**b**

**a**

**Transcript Positions**

**100 bp marker**

**300 bp marker**

**Digestion product**

**1-**

**2-**

**2+**

**3-**

**3+**

**4-**

**4+**

**1+**
