# Supplementary material for: Genetic regulation of MUC1 alternative splicing in human tissues
Source: Br J Cancer. 2008 Aug 26;99(6):978–85. doi: 10.1038/sj.bjc.6604617 (PMC2538764; doi:10.1038/sj.bjc.6604617)
Supplement: Supplementary Table 1, Supplementary Information and Supplementary Figure 1 Legend [file 6604617x2.doc]

Supplementary Information for electronic publication

MUC1 transcripts available on the sequence databases

The **a** variant is represented by eight transcripts (AY327584, AY327586, AY327587, AY327599, BC120975, M31823, M32739, S81781) and the **b** transcripts by 26 sequences (AF348143, AY327582, AY327583, AY327585, AY327588, AY327589, AY327590, AY327591, AY327592, AY327595, AY327596, AY327597, AY327598, AY327600, AY466157, BC120974, J05581, J05582, M32738, S81736, U60259, U60260, U60261, X52228, X52229, X80761).

Supplementary Table 1.

Primer sequences and PCR conditions used. The Cy5 labelled primer is indicated. Primers used as control for expression levels also shown

| Product | Primer sequence (5’ to 3’) | Annealing Temp. Used | Cycles |
| --- | --- | --- | --- |
| *MUC1* rs4072037 AG SNP Genotyping | Cy5-CTGGGACCGAGGTGACATCC cctaaacccgcaacagttgttac | 62°C | 34 |
| *MUC1* **a**  **b c d** detection | Cy5-CAGTCTCCTTTCTTCCTGCTG  TACGCTGCTGGTCATACTCAC | 59°C | 30 |
| *MUC1* minus TR alternative splicing detection | Cy5-CAGTCTCCTTTCTTCCTGCTG  CTCTGCAGCTCTTGGTAGTAGTC | 59°C | 30-32 |
| MUC1 exon 5 to 6 detection | GCCATTTCCTTTCTCTGCCC  GCTACGATCGGTACTGCTAG | 52°C | 30 |
| RPS14 control primers | GGCAGACCGAGATGAATCCTCA  CAGGTCCAGGGGTCTTGGTCC | 65°C | 26-30 |

Legend to Supplementary Figure 1 *AlwN1* digested RT-PCR products from individuals of known genotype. The first two samples are from AA (1) and GG (2) homozygotes. The third sample is from a typical heterozygote, while the fourth is from a heterozygote in which very little a transcript is detected. Both undigested (-) and digested (+) samples are shown. Digestion products are marked with an arrow. Sample 2 was from fetal stomach, all other samples from fetal lung. When transcript b from the AA homozygote is cut using the *AlwNI* the transcript b peak disappears after digestion and an extra shorter fragment is generated (marked on Figure as “Digestion Product”). In contrast there is no evidence of enzyme digestion of the a transcript from the GG homozygote. In the typical heterozygote, the b transcript is cut, while the a transcript is not (Figure 4). In the very asymmetric heterozygote, the minor a transcript is as expected unchanged, and the b transcript is digested just as expected.
